# Canine heartworm disease (*Dirofilaria immitis*) in Western Europe: survey of veterinary awareness and perceptions

**DOI:** 10.1186/1756-3305-7-206

**Published:** 2014-04-29

**Authors:** Claudio Genchi, Dwight Bowman, Jason Drake

**Affiliations:** 1 Department of Veterinary Science and Animal Health, Università degli Studi di Milano, via Celoria 10, 20133 Milano, Italy; 2 Department of Microbiology and Immunology, College of Veterinary Medicine, Cornell University, Ithaca, NY 1483, USA; 3 Novartis Animal Health US, Inc., 3200 Northline Ave, Suite 300, Greensboro, NC 27408, USA

**Keywords:** Heartworm, *Dirofilaria immitis*, Dog, Veterinary awareness, Europe

## Abstract

**Background:**

This survey examines the experience and opinion of veterinarians with canine heartworm (HW; *Dirofilaria immitis*) infection in non-endemic and endemic areas of Europe.

**Methods:**

A questionnaire was distributed by e-mail to veterinary practitioners within non-endemic countries including UK, Germany, and Netherlands,non-endemic regions of France, Spain, Italy and endemic regions of France, Spain and Italy. The main questions were about the HW cases seen in the last 12 months, if the number of cases had changed over the last 5 years and practitioner awareness of the ESCAAP Guidelines on HW. Additional questions examined practitioner perception regarding the risk of HW for pets travelling in endemic areas and the use of preventatives including veterinary perception of pet owner compliance.

**Results:**

Overall 584 responses, 389 from non-endemic countries and regions and 195 from endemic regions were obtained. Most of the cases were seen in endemic regions, although in Germany 20% of veterinarians reported cases of HW infection. Overall, 10% of practitioners in non-endemic areasand 12% in endemic areas, respectively, reported an increasing number of cases. The practitioner awareness of ESCCAP guidelines is low. Veterinarians responded that preventative drugs were prescribed to most client dogs in endemic areas and most practitioners rated owner compliance as good or excellent in following the veterinarian’s guidance for HW prevention. Overall 63% of veterinarians responded that owners are more or less aware of the HW risk traveling with their dogs in endemic areas. Forty four percent of responders in non-endemic regions considered it somewhat likely to very likely that HW infection will become endemic in their regions.

**Conclusions:**

In both non-endemic and endemic countries at least 1 responding practitioner admitted seeing a case of canine HW infection and has shown awareness regarding infection. Ten percent of practitioners stated that the number of cases is increasing. Although the number of owners traveling with their dogs was low, the perception of surveyed veterinarians was that owners were quite aware of the HW risk for their dogs. Several veterinary practitioners believe that HW infection could become endemic in their non-endemic area within the next 10 years.

## Background

Historically, canine heartworm (HW, *Dirofilaria immitis*) infection in Europe was localized in the southern areas and until the middle of the 1980’s the most endemic area was northern Italy [1], where the parasite was first observed by Birago in 1626 [2] in a greyhound dog at autopsy. However, there has been significant debate over the last few years regarding the risk of spread of dirofilarial parasites to non-endemic European areas as a consequence of changing climate, dog movement and relocation [3-5]. Currently, HW infection has been reported from non-endemic areas of both central and southern Italy [6-8] and in some northern and north-eastern European countries such as Czech Republic, Hungary, Serbia, Slovak Republic, Switzerland [4,9], and recently in Romania [10] and Poland [11]. Furthermore, *D. immitis* larvae have been found in mosquitoes in Germany [12].

People frequently travel with their pets between non-endemic and endemic areas. If travelling dogs become HW infected, they can act as donors of microfilariae to local mosquito species making spread of the infection to naïve canine population possible. Heartworm infection in dogs is a chronic condition and most infected dogs do not show any symptoms for months or years, depending on the worm burden [13].

This survey was conducted to examine the opinions and experiences of veterinarians with canine HW infection in both non-endemic and endemic areas as well as their awareness of European Scientific Counsel Companion Animal Parasites Guidelines on prevention of HW in Europe [14]. This is the first survey that has tried to assess the condition throughout Europe, not just in individual countries. The survey was not all inclusive, but it is a major start in obtaining information from practitioners as to where they believe heartworm is located.

## Methods

An online survey was developed and provided to companion animal veterinarians from June through September 2013 across *D. immitis* non-endemic countries including United Kingdom, Germany and Netherlands, non-endemic regions of France, Spain, Italy and endemic regions of France, Spain and Italy. Over 5382 questionnaires, with a minimum of 300 questionnaires per country, were email distributed to veterinary clinics with the goal of obtaining at least 100 responses per country. The clinics were targeted through a combination of purchased veterinary email lists as well as email addresses that Novartis had accumulated independently from veterinarians with the goal of communicating to as many veterinarians as possible. In addition, 1010 flyers with links to the online survey were printed and delivered to veterinary clinics in Italy, Spain and France to encourage participation. Two sets of questionnaires were used for non-endemic or endemic regions. The questionnaires were available in English, German, Dutch, French, Spanish and Italian. Depending on response rates, a reminder was sent out 2 weeks after the first email. The main questions for practitioners working in both non-endemic and endemic areas were:

– Over the last 12 months, how many cases of HW infection have you seen at your clinic?

– How have the number of HW cases changed over the last 5 years?

– How aware are you of the ESCCAP guidelines on HW infection?

Further questions were posed to veterinarians working in endemic areas about the use of HW preventative drugs and the compliance of animal owners in following guidance regarding HW prevention. In non-endemic regions, additional questions were about the percentage of dog owners travelling with their pets in endemic regions, the perception of veterinarians and owners of the risk of HW infection being brought into non-endemic regions by dogs travelling in endemic areas, and how likely did the veterinarians think it might be that HW will become endemic within the next 10 years in their region/country.

## Results

A total of 584 responses, representing a response rate of approximately 9.1%, were obtained including 389 from non-endemic areas (UK: 33, Germany: 101, Netherlands: 34; non-endemic areas of France: 86, Spain: 68, Italy: 67) and 195 from endemic areas (France: 47, Spain: 41, and Italy: 107).

Twenty-five percent of responders in non-endemic regions had seen one or more cases over the last 12 months. Ten percent reported seeing 2-9 cases over the past 12 months. More than 10 cases were not reported by responders from any of the non-endemic countries (UK, Germany and Netherlands). Twenty three percent of veterinary practitioners in Germany (23/101) reported 2-9 cases, 4 (2%, 4/221) reported more than 10 cases in non-endemic areas of France, Italy and Spain (Table 1). In endemic regions, 32% (15/47), 85% (35/41) and 58% (62/107) of veterinary practitioners from France, Spain and Italy, respectively, reported cases of HW infection in the last 12 months. Two veterinary practitioners in France and in Spain, and one in Italy, equivalent to 2.6% of responders from endemic areas, reported more than 26 cases. Eleven to 25 cases were seen by 2 veterinary practitioners in France, 3 in Spain and 1 in Italy (Table 2).

**Table 1:** **Number of cases of canine heartworm ( *Dirofilaria immitis *) infections: non-endemic regions seen by the practitioners in the last 12 months**

	**Number of cases: non-endemic countries/regions**					
**Country**	**0**	**1**	**2–5**	**6–9**	**>10**	**Responders**
UK	30	1	2	0	0	33
Germany	40	38	20	3	0	101
Netherlands	32	1	1	0	0	34
France	80	4	1	0	1	86
Spain	57	7	1	1	2	68
Italy	51	10	5	0	1	67
Total (%)	290 (74)	61 (16)	30 (8)	4 (1)	4 (1)	389

**Table 2:** **Number of cases of canine heartworm ( *Dirofilaria immitis *) infections: endemic regions seen by the practitioners in the last 12 months**

	**Number of cases: endemic regions**					
	**Number clinics responding**					
**Country**	**0**	**1–5**	**6–10**	**11–25**	**>26**	**Responders**
France	32	10	1	2	2	47
Spain	6	21	9	3	2	41
Italy	45	58	2	1	1	107
Total (%)	83 (42)	89 (46)	12 (6)	6 (3)	5 (3)	195

Overall, 34% (62/584) of practitioners reported an increasing number of HW cases. In non-endemic countries,14% (23/168) reported an increase, 2% (3) a decrease and 24% (40) no change (Table 3); although in Germany, 20% of veterinarians (20/101) reported an increasing number of cases. Overall, in non-endemic regions of France, Spain and Italy, 7% (16/221) of veterinarians reported an increase of HW cases, 1.3% (3) a decrease and 18% (39) no change (Table 3). The highest increase of cases was reported from non-endemic Italy (13/67, 19%). In endemic regions of France, Spain and Italy, overall 9% of responders (18/195) reported an increase of HW cases, 29% (56) a decrease and 25% (49) no change. The highest number of increasing cases was reported from Spain (11/41, 27%; Table 3).

**Table 3:** **Change in number of cases of canine heartworm ( *Dirofilaria immitis *) infections over the last 5 years**

	**Clinics reporting changes in number of cases**				
**Country**	**Increase in cases (%)**	**Decrease in cases (%)**	**No change in cases (%)**	**No cases (%)**	**Total responders (%)**
UK (non-endemic)	2 (5)	0 (0)	1 (1)	30 (11)	33 (9)
Germany (non-endemic)	20 (51)	3 (50)	38 (48)	40 (15)	101 (26)
Netherlands (non-endemic)	1 (3)	0 (0)	1 (1)	32 (12)	34 (9)
France (non-endemic)	1 (3)	2 (33)	7 (9)	76 (29)	86 (22)
France (endemic)	5 (22)	4 (7)	9 (18)	29 (43)	47 (24)
Spain (non-endemic)	2 (5)	1 (17)	18 (23)	47 (18)	68 (17)
Spain (endemic)	11 (48)	11 (20)	14 (29)	5 (8)	41 (21)
Italy (non-endemic)	13 (33)	0 (0)	14 (18)	40 (15)	67 (17)
Italy (endemic)	7 (30)	41 (73)	26 (53)	33 (49)	107 (55)
Total non-endemic	39	6	79	265	389
Total endemic	23	56	49	67	195

Most veterinary practitioners from both non-endemic and endemic areas were not aware of ESCCAP HW guidelines (317/584, 54%) and only 9% were quite or very aware of the guidelines (54/389, 9%) (Table 4). However, 54% of veterinarians from endemic regions of Spain (22/41) and 30% from endemic regions of Italy (30/107) reported to be moderately-very aware about the guidelines and 41% (17/41) and 30% (32/107), respectively, declared that guidelines are well implemented into their clinical practice.

**Table 4:** **How aware are the veterinary practitioners of the ESCCAP Heartworm ( *Dirofilaria immitis *) Guidelines**

	**Awareness of ESCCAP Heartworm Guidelines**					
	**Number clinics responding (% responding)**					
**Country**	**Not aware**	**Slightly aware**	**Moderately aware**	**Quite aware**	**Very aware**	**Responders**
UK (non-endemic)	22 (67)	5 (15)	3 (9)	3 (9)	0 (0)	33
Germany (non-endemic)	40 (40)	38 (38)	20 (20)	3 (3)	0 (0)	101
Netherlands (non-endemic)	32 (94)	1 (3)	1 (3)	0 (0)	0 (0)	34
France (non-endemic)	63 (73)	9 (10)	10 (12)	2 (2)	2 (2)	86
France (endemic)	29 (62)	9 (19)	4 (9)	4 (9)	1 (2)	47
Spain (non-endemic)	25 (37)	13 (19)	22 (32)	6 (9)	2 (3)	68
Spain (endemic)	15 (37)	4 (10)	10 (24)	9 (22)	3 (7)	41
Italy (non-endemic)	35 (52)	11 (16)	14 (21)	5 (7)	2 (3)	67
Italy (endemic)	56 (52)	19 (18)	20 (19)	8 (7)	4 (4)	107
Total (non-endemic)	217 (56)	77 (20)	70 (18)	19 (5)	6 (2)	389
Total (endemic)	100 (51)	32 (16)	34 (17)	21 (11)	8 (4)	195
Total	317 (54)	109 (19)	104 (18)	40 (7)	14 (2)	584

Overall, 12% of veterinarians in endemic regions did not prescribe a preventative treatment against HW infection over the last 12 months. It was prescribed by 36% of responders to all the dogs in their practice while from 5% to 25% of responders prescribed preventatives to different percentages of dogs (from less than 25% to 99%) (Table 5). One hundred and forty (74%) veterinarians rated owner compliance in following the guidance for HW prevention to be excellent or good, 19% reasonable and 9% poor or non-compliant (Table 6).

**Table 5:** **Over the last 12 months, approximately what percentage of dogs in your clinic within an endemic area, have been prescribed preventative treatment against heartworm ( *Dirofilaria immitis *)?**

	**Percentage of dogs prescribed heartworm preventative within a clinic in an endemic area**						
	**Number clinics responding (% responding)**						
**Country**	**None**	**≤25%**	**26–50%**	**51–75%**	**76–99%**	**All**	**Responders**
France	24 (51)	9 (19)	2 (4)	9 (19)	2 (4)	1 (2)	47
Spain	0 (0)	8 (20)	6 (15)	5 (12)	13 (32)	9 (22)	41
Italy	0 (0)	3 (3)	2 (2)	6 (6)	35 (33)	61 (57)	107
Total	24 (12)	20 (10)	10 (5)	20 (10)	50 (26)	71 (36)	195

**Table 6:** **How do you rate the compliance of companion animal owners that live in your endemic area in following guidance regarding heartworm ( *Dirofilaria immitis *) prevention in their dogs?**

	**Veterinary rating of pet owner compliance in endemic regions**					
	**Number clinics responding (% responding)**					
	**Excellent**	**Good**	**Reasonable**	**Poor**	**Non-compliant**	**Responders**
France	10 (21)	7 (15)	19 (40)	4 (9)	7 (15)	47
Spain	0 (0)	21 (51)	17 (41)	3 (7)	0 (0)	41
Italy	26 (24)	76 (71)	2 (2)	3 (3)	0 (0)	107
Total	36 (18)	104 (53)	38 (19)	10 (5)	7 (4)	195

The percentage of dog owners that had traveled from non-endemic to endemic countries/regions estimated by the veterinary practitioners is shown in Table 7. The percentages between 0-10% and 11%-25% account for the higher frequencies (40% and 34%, respectively), while percentages between 51%-75% and 76%-100% account for 6% and 1% only, respectively (Table 7). In non-endemic regions, Fifty three percent of veterinary practitioners (207/389) consider that there is no or little risk of introduction of HW infection through traveling dogs, 41% (163/389) declare some or moderate risk and 5% high risk (19/389; Table 8). Thirty seven percent (142/389) of practitioners think that owners are not aware of the risk of HW when they travel with their dogs in endemic areas, 39% (153) think the owners are slightly aware, 23% (89) think the owners are somewhat or moderately aware and only 1% (5) think that owners are very aware (Table 9).

**Table 7:** **What percentage of companion animal owners from your non-endemic country/region do you think travel with their pets to heartworm ( *Dirofilaria immitis *) endemic areas such as Spain, south of France, Italy, Greece, Turkey and other countries along the Adriatic coast?**

	**Percentage of pet owners that travel with pets from non-endemic regions to endemic regions**					
	**Number clinics responding (% responding)**					
**Country**	**0–10%**	**11–25%**	**26–50%**	**51–75%**	**76–100%**	**Responders**
UK	21 (64)	8 (24)	4 (12)	0 (0)	0 (0)	33
Germany	29 (29)	39 (39)	26 (26)	6 (6)	1 (1)	101
Netherlands	15 (44)	13 (38)	2 (6)	4 (12)	0 (0)	34
France	38 (44)	34 (40)	10 (12)	4 (5)	0 (0)	86
Spain	26 (38)	21 (31)	17 (25)	4 (6)	0 (0)	68
Italy	25 (37)	19 (28)	15 (22)	6 (9)	2 (3)	67
Total	154 (40)	134 (34)	74 (19)	24 (6)	3 (1)	389

**Table 8:** **What is your perceived risk of heartworm ( *Dirofilaria immitis *) infection being brought back into your non-endemic country/region by traveling dogs**

	**Perceived risk of heartworm when traveling with pets return to non-endemic areas from endemic areas**						
	**Number clinics responding (% responding)**						
**Country**	**No risk**	**Little risk**	**Some risk**	**Moderate risk**	**High risk**	**Total risk**	**Responders**
UK	2 (6)	17 (52)	13 (36)	1 (3)	0 (0)	31 (94)	33
Germany	8 (8)	29 (29)	34 (34)	22 (22)	8 (8)	93 (92)	101
Netherlands	3 (9)	12 (35)	15 (44)	4 (12)	0 (0)	31 (91)	34
France	13 (15)	59 (69)	11 (13)	2 (2)	1 (1)	73 (85)	86
Spain	12 (18)	30 (44)	17 (25)	6 (9)	3 (4)	56 (82)	68
Italy	6 (9)	16 (24)	25 (37)	13 (19)	7 (10)	61 (91)	67
Total	44 (11)	163 (42)	115 (30)	48 (12)	19 (5)	345 (89)	389

**Table 9:** **How do you think companion animal owners are aware of the risk of heartworm ( *Dirofilaria immitis *) when traveling to heartworm endemic areas?**

	**Perceived pet owner awareness of heartworm risk when traveling from non-endemic areas to endemic areas**					
	**Number clinics responding (% responding)**					
**Country**	**Not aware**	**Slightly aware**	**Somewhat aware**	**Moderately aware**	**Very aware**	**Responders**
UK	16 (48)	14 (42)	2 (6)	1 (3)	0 (0)	33
Germany	35 (35)	49 (49)	13 (13)	3 (3)	1 (1)	101
Netherlands	8 (24)	16 (47)	4 (12)	5 (15)	1 (3)	34
France	46 (53)	29 (34)	10 (12)	1 (1)	0 (0)	86
Spain	21 (31)	21 (31)	17 (25)	6 (9)	3 (4)	68
Italy	16 (24)	24 (36)	18 (27)	9 (13)	0 (0)	67
Total	142 (37)	153 (39)	64 (16)	25 (6)	5 (1)	389

Table 10 shows the perception of veterinarians in non-endemic countries/areas that HW should become endemic in their regions in the next 10 years. Twenty three percent think that this is not likely, 33% that it is slightly, 38% that it is somewhat or moderately likely and 6% that it is very likely.

**Table 10:** **How likely do you think that heartworm ( *Dirofilaria immitis *) will become endemic in your currently non-endemic region within the next 10 years?**

	**Perceived risk of heartworm spreading to non-endemic areas**					
	**Number clinics responding (% responding)**					
**Country**	**Not at all likely**	**Slightly likely**	**Somewhat likely**	**Moderately likely**	**Very likely**	**Responders**
UK	11 (33)	12 (36)	4 (12)	5 (15)	1 (3)	33
Germany	18 (18)	30 (30)	28 (28)	23 (23)	2 (2)	101
Netherlands	3 (9)	17 (50)	7 (21)	6 (18)	1 (3)	34
France	34 (40)	37 (43)	10 (12)	2 (2)	3 (3)	86
Spain	20 (29)	22 (32)	17 (25)	5 (7)	4 (6)	68
Italy	3 (4)	12 (18)	25 (37)	16 (24)	11 (16)	67
Total	89 (23)	130 (33)	91 (23)	57 (15)	22 (6)	389

## Discussion

As is true for many surveys, participation was entirely voluntary which may lead to some bias in responses. Individuals with an interest in the topic are more likely to participate. Although the goal of 100 responders was not achieved in all the countries included in the survey, the overall number of responders is sufficient to draw some conclusions.

In all countries, at least one veterinary practitioner has seen at least one case of canine HW infection in the last 12 months. Nevertheless, while the number of responders from non-endemic countries such as UK and Netherland is low (33 and 34, respectively), probably because the cases are quite rare and diagnosed in imported or relocated dogs [15,16], the number of responders from Germany was quite significant, confirming the empirical data that HW infection is quite frequently diagnosed in travelling or relocated dogs [4]. Furthermore, the spreading of autochthonous cases of *D. repens*, the agent of subcutaneous dirofilariosis [16], has alerted German practitioners to *Dirofilaria* infections. As expected, fewer veterinarians from non-endemic regions reported cases than veterinarians from endemic regions, although the number of veterinarians reporting cases in Germany (61 cases) is the highest for non-endemic areas including France, Spain and Italy where the infection is endemic in many areas. Furthermore, the percent of practitioners that declared an increasing number of cases is higher in non-endemic areas than in endemic ones, confirming the spread of infection. Most cases were seen in the last 5 years in Germany, in non-endemic areas of Italy and in endemic areas of Spain possibly as a consequence of changing climate, confirming the provisional expansion of the risk to naïve areas [17,18]. In endemic regions, probably more than 357 cases (89 responder at 1-5 cases, 12 at 6-10 cases, 6 at 11-25 cases, and 5 at 26 or more cases) of heartworm in the last 12 months were reported by veterinary practitioners in France, Spain and Italy, confirming that in spite of the use of preventative drugs, the infection is still frequent. Of note is that the number of practitioners prescribing preventative drugs to all their clients’ dogs is quite low in the endemic areas of France and Spain. Only in Italy are more than half of the responding veterinarians prescribing preventative drugs to all their clients’ dogs and this may be a potential reason for the consistent decrease in cases seen in the country.

Overall, the awareness of ESCCAP HW guidelines is low, although veterinary practitioners in endemic regions are, on average, over twice as aware compared with colleagues in non-endemic regions, but quite a few claim to have implemented the guidelines into their clinical practice. Nevertheless, the perception of owner compliance in following the practitioners’ guidance regarding HW prevention in their dogs is good or excellent in most endemic countries.

Regarding the movement of dogs, only limited pet travel was noted amongst respondents. More than 60% of respondents stated that less than 25% of pets attending their clinics would likely travel to an endemic HW region. For instance, from the UK, most veterinary practitioners have stated that no pets would travel to endemic areas. On the other hand, 50% of practitioners from non-endemic Italy and Germany declare there is some risk to a high risk that HW could be introduced by dogs traveling in endemic areas, and more than 50% of responders consider that owners are aware of the infection risk (from slight to very aware). This data on owner compliance in following veterinary guidance in HW shows a good degree of knowledge about and attention to animal welfare by owners regarding this severe and potentially life-threatening disease. Finally, the perception that HW infection could become endemic in naïve areas is quite high in Italy and Germany.

## Conclusion

This survey gives an overview of the current experience and opinions of European veterinary practitioners on HW infection both from non-endemic and endemic countries. The general perception is that the number of cases is increasing. Although the percent of owners traveling with their dogs is relatively low, the perception is that they are quite aware of the HW risk for their dogs. More than one third of veterinary practitioners have stated that HW infection could become endemic in non-endemic areas within the next 10 years.

## Competing interest

JD, is an employee of Novartis Animal Health. CG and DDB have no competing financial interests to disclose.

## Authors’ contributions

JD prepared the questionnaires that were critically examined and approved by CG and DB. JD sent the questionnaires to veterinary practitioners. All the authors contributed equally in examining the results and preparing the manuscript. All the authors read and approved the final version of the manuscript.

## References

[B1] Genchi C Solari Basano F Marone RV Petruschke G Seward RLS, Batavia IL Canine and Feline Heartworm in Europe with special emphasis to Italy Proceedings of the heartworm symposium '98, Tampa, Florida, USA, 1-3 May, 1998 1998 75 82

[B2] Birago F Trattato Cinegetico, Ovvero della Caccia 1626 Milano, Italy: V. Sfondato 77

[B3] Genchi C Rinaldi L Cascone C Mortasino M Cingoli G Is heartworm disease really spreading in Europe? Vet Parasitol 2005 133 137 148 10.1016/j.vetpar.2005.04.009 15885913

[B4] Pantchev N Etzold M Daugschies A Dyachenko V Diagnosis of imported canine filarial infections in Germany 2008–2010 Parasitol Res 2011 109 Suppl 1 S61 S76 21739376 10.1007/s00436-011-2403-7

[B5] Sassnau R Genchi C Qualitative risk assessment to the endemisation of Dirofilaria repens in the state of Brandenburg (Germany) based on temperature-dependent vector competence Parasitol Res 2013 112 2647 2652 10.1007/s00436-013-3431-2 23609600

[B6] Mortarino M Musella V Costa V Genchi C Cingoli G Ranaldi L GIS modeling for canine dirofilariosis risk assessment in central Italy Geosp Health 2008 2 253 261 10.4081/gh.2008.248 18686273

[B7] Otranto D Capelli G Genchi C Changing distribution patterns of canine vector borne diseases in Italy: leishmaniosis vs. dirofilariosis Parasit Vectors 2009 2 (Suppl 1) S2 http://www.parasitesandvectors.com/content/2/S1/S2 10.1186/1756-3305-2-S1-S2 PMC2679394 19426441

[B8] Giangaspero A Marangi M Latrofa MS Martinelli D Traversa D Otranto D Genchi C Evidences of increasing risk of dirofilarioses in southern Italy Parasitol Res 2013 112 1361 1375 10.1007/s00436-012-3206-1 23224639

[B9] Otranto D Dantas-Torres F Brianti E Traversa D Petrić Genchi C Capelli G Vector-borne helminths of dogs and humans in Europe Parasit Vectors 2013 6 6 15 10.1186/1756-3305-6-6 23324440 PMC3564894

[B10] Mircean V Dumitrache MO Györke A Pantchev N Jodies R Mihalca AD Cozma V Seroprevalence and geographic distribution of Dirofilaria immitis and tick-borne infections (Anaplasma phagocytophilum, Borrelia burgdorferi sensu lato, and Ehrlichia canis) in dogs from Romania Vector Borne Zoonotic Dis 2012 12 595 604 10.1089/vbz.2011.0915 22607068

[B11] Świątalska A Demiaszkiewicz AW First autochthonous case of Dirofilaria immitis invasion in dog in Poland Žycie Weterynaryjne 2012 87 685 686 [in Polish]

[B12] Kronefeld M Kampen H Sassnau R Werner D Molecular evidence for the occurrence of *Dirofilaria immitis*, *Dirofilaria repens* and *Setaria tundra* in mosquitoes from Germany Parasit Vectors 2014 7 30 http://www.parasitesandvectors.com/content/7/1/30 10.1186/1756-3305-7-30 24433279 PMC3898823

[B13] McCall JW Genchi C Kramer LH Guerrero J Venco L Heartworm disease in animals and humans Adv Parasitol 2008 66 193 285 18486691 10.1016/S0065-308X(08)00204-2

[B14] ESCCAP GL5 2012 http://www.esccap.org/uploads/docs/ih38c2d6_ESCCAP_Guidelines_GL5_01Oct2012.pdf

[B15] Thomas RE A case of canine heartworm disease (Dirofilaria immitis) in the UK Vet Rec 1985 117 14 15 10.1136/vr.117.1.14 3895715

[B16] Meyer HP Wolvekamp P van Maanen C Stokof AA Seven cases of heartworm disease (dirofilariosis) in dogs in The Netherlands Vet Q 1994 16 169 174 10.1080/01652176.1994.9694443 7871703

[B17] Sassnau R Kohn M Demeler J Kohn B Müller E Krücken J von Samson-Himmelstjerna G Is Dirofilaria repens Endemic in the Havelland District in Brandenburg, Germany? Vector Borne Zoonotic Dis 2013 Aug 6. [Epub ahead of print] 10.1089/vbz.2012.1293 23919602

[B18] Genchi C Mortarino M Rinaldi L Cringoli G Traldi G Genchi M Changing climate and changing vector-borne disease distribution: the example of Dirofilaria in Europe Vet Parasitol 2011 176 295 299 10.1016/j.vetpar.2011.01.012 21300439

